# Precision Adverse Drug Reactions Prediction with Heterogeneous Graph Neural Network

**DOI:** 10.1002/advs.202404671

**Published:** 2024-12-04

**Authors:** Yang Gao, Xiang Zhang, Zhongquan Sun, Payal Chandak, Jiajun Bu, Haishuai Wang

**Affiliations:** ^1^ Department of Hepatobiliary and Pancreatic Surgery The Second Affiliated Hospital Zhejiang University School of Medicine Hangzhou 310009 China; ^2^ College of Computer Science Zhejiang University Hangzhou 310058 China; ^3^ Department of Computer Science The University of North Carolina at Charlotte Charlotte NC 28223‐0001 USA; ^4^ Harvard‐MIT Health Sciences and Technology Cambridge MA 02139 USA; ^5^ Shanghai Artificial Intelligence Laboratory Shanghai 200232 China

**Keywords:** adverse drug reactions, FDA adverse event reporting system (FAERS), graph neural network, precision medicine

## Abstract

Accurate prediction of Adverse Drug Reactions (ADRs) at the patient level is essential for ensuring patient safety and optimizing healthcare outcomes. Traditional machine learning‐based methods primarily focus on predicting potential ADRs for drugs, but they often fall short of capturing the complexity of individual demographics and the variations in ADRs experienced by different people. In this study, a novel framework called Precise Adverse Drug Reaction (PreciseADR) for patient‐level ADR prediction is proposed. The approach effectively integrates relations between patients and ADRs, and harnesses the power of heterogeneous Graph Neural Networks (GNNs) to address the limitations of traditional methods. Specifically, a heterogeneous graph representation of patients is constructed, encompassing nodes that represent patients, diseases, drugs, and ADRs. By leveraging edges in the graph, crucial connections are captured such as a patient being affected by diseases, taking specific drugs, and experiencing ADRs. Next, a GNN‐based model is utilized to learn latent representations of the patient nodes and facilitate the propagation of information throughout the graph structure. By employing patient embeddings that consider their diseases and drugs, potential ADRs can be accurately predicted. The PreciseADR is dedicated to effectively capturing both local and global dependencies within the heterogeneous graph, allowing for the identification of subtle patterns and interactions that play a significant role in ADRs. To evaluate the performance of the approach, extensive experiments are conducted on a large‐scale real‐world healthcare dataset with adverse reports from the FDA Adverse Event Reporting System (FAERS). Experimental results demonstrate that the PreciseADR achieves superior predictive performance in identifying patient‐level ADRs, surpassing the strongest baseline by 3.2% in AUC score and by 4.9% in Hit@10.

## Graphical abstract

PreciseADR leverages heterogeneous Graph Neural Networks (GNNs) to predict patient‐level Adverse Drug Reactions (ADRs) by integrating relationships between patients, diseases, drugs, and ADRs. This novel framework captures complex patient demographics and ADR variations, outperforming traditional methods. Extensive experiments on the FDA Adverse Event Reporting System (FAERS) dataset show a 3.2% AUC score improvement and a 4.9% increase in Hit@10.

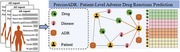

## 1 Introduction

Adverse Drug Reactions (ADRs)^[^ ^1^, ^2^, ^3^, ^4^, ^5^ ^]^ encompass undesired or detrimental effects resulting from the administration of medical products. The U.S. Food and Drug Administration (FDA) provides alarming statistics,^[^ ^6^ ^]^ estimating that annually, over 2 216 000 severe ADRs occur in hospitalized patients, leading to more than 106 000 deaths. Moreover, statistical data^[^ ^7^ ^]^ indicates that addressing the wide range of public health issues associated with ADRs in the United States incurs annual expenditures amounting to 30.1 billion dollars. The prediction of potential ADRs not only holds significant importance in drug development^[^ ^8^, ^9^ ^]^ and precision medicine^[^ ^10^ ^]^ but also alleviates the clinical and economic burden on the government. In the healthcare domain, the monitoring and understanding of ADRs are crucial and facilitated by adverse event reporting systems, such as the Food and Drug Administration Adverse Event Reporting System (FAERS),^[^ ^11^ ^]^ the Japanese Adverse Drug Event Report (JADER),^[^ ^12^ ^]^ and the Canada Vigilance Program.^[^ ^13^ ^]^ These systems serve as essential repositories for data collection and analysis, providing invaluable support to healthcare professionals, researchers, and regulatory authorities in identifying safety concerns, assessing risks and benefits, and improving drug safety profiles.

In recent years, there has been a discernible shift toward the adoption of machine learning‐based methodologies^[^ ^14^, ^15^, ^16^, ^17^, ^18^, ^19^, ^20^, ^21^ ^]^ for inferring potential drug side effects with drug features. Recent research endeavors have further advanced this domain by introducing sophisticated neural network architectures, such as graph neural networks, to leverage the structured chemical descriptions of drugs for ADR prediction.^[^ ^22^, ^23^, ^24^, ^25^ ^]^ Those data‐driven methods have demonstrated a significant advantage in estimating drug ADRs and have become integral to in vitro toxicity testing and widespread application in drug development. Nevertheless, a prevalent limitation in existing approaches pertains to their capacity to predict general drug side effects while falling short of delivering patient‐level predictions. To overcome these challenges, novel datasets and approaches are necessary to enhance prediction accuracy and enable tailored interventions that account for diverse patient characteristics and their unique susceptibility to ADRs.

In this study, we introduced a novel patient‐level ADR prediction framework, referred to as PreciseADR, aimed at better serving the field of precision medicine. Specifically, we identify ADRs associated with gender and age through a statistical analysis of adverse event reports from FAERS. This discovery underscores the significance of comprehensively considering individual patient profiles, including medical histories and demographic information, in predicting potential ADRs related to drug combinations. In response to this observation, we introduced a novel patient‐level ADR prediction framework, PreciseADR. Specifically, we proposed the PreciseADR framework, based on graph neural networks, to capture latent relationships between patients, diseases, drugs, and ADRs, thereby improving the understanding of patient representations and achieving patient‐level ADR predictions. Through comparative analysis with state‐of‐the‐art baselines, including traditional machine learning algorithms and graph‐based models, we demonstrated the superior performance of our approach in patient‐level ADR prediction. Furthermore, our experiments highlighted the pivotal role of patient‐specific attributes such as age and gender in ADR prediction. These analytical findings not only enhance our comprehension of ADRs but also aid healthcare professionals in making informed decisions. The main contributions of our work are summarized as follows:

1. We identified gender‐ and age‐related ADRs through statistical analyses, highlighting the significance of incorporating patient‐specific information for precision ADR prediction.
2. We made the first effort on the problem of patient‐level ADR prediction, using patient‐specific information for personalized ADR prediction.
3. We introduced a patient‐level ADR prediction framework, PreciseADR, aimed at learning latent patient representations to enhance the precision of ADR predictions. PreciseADR achieves this by aggregating information from patients' physiological characteristics, medical histories, medication records, and ADRs.
4. We test PreciseADR on real‐world datasets. Experimental results strongly support the effectiveness of our method, demonstrating a significant performance improvement compared to conventional methods.

## 2 Results

### 2.1 Overview of the PreciseADR Approach

**Figure 1:**
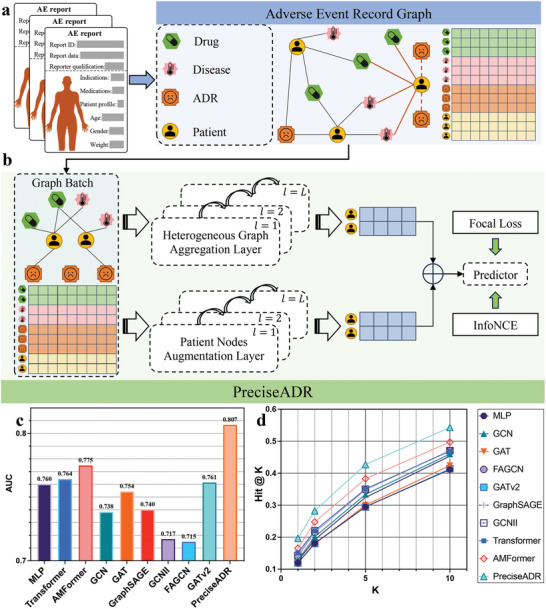
Overview of the Precise Adverse Drug Reaction prediction‐framework (PreciseADR). By leveraging the intricate relationships among patients and ADRs, PreciseADR demonstrates superior performance compared to all baselines. a) A demonstration of the Adverse Event Report (AER) Graph using FAERS data. Nodes represent diseases, medications, ADRs, and patients, with edges capturing patient associations with specific diseases and medications. When predicting ADRs for new patients, they are added to the AER Graph and linked to their known diseases and candidate drugs. b) The framework of the PreciseADR. PreciseADR employs a heterogeneous graph neural network to extract diverse information from the AER Graph. It enhances patient representations with contrastive learning techniques. c) and d) PreciseADR's exceptional performance in patient‐level ADR prediction on the PLEASE dataset, derived from adverse reports within FAERS.

**Figure** **1** provides an overview of the PreciseADR framework, representing a foundational framework of patient‐level ADR prediction research. We have introduced a heterogeneous graph known as the “Adverse Event Report Graph” (AER Graph) to capture the intricate relationships among patients and adverse drug reactions. In the AER Graph, nodes are assigned to diseases, drugs, ADRs, and patients, with edges created when patients are associated with diseases, take specific drugs, or experience ADRs, as depicted in Figure 1a. Importantly, it is worth noting that all edges within the AER Graph originate from existing adverse event reports. For new patients requiring ADR predictions, their information is added to the AER Graph, linking them to their known diseases and the candidate drugs, and the PreciseADR will further predict the potential ADRs. This expansion of the graph allows us to incorporate specific patient data for ADR prediction, facilitating a more comprehensive understanding of the ADR landscape and the integration of diverse information sources.

Furthermore, PreciseADR employs a model based on heterogeneous graph neural networks (HGNNs) to comprehensively analyze the AER Graph for precise ADR prediction, as illustrated in Figure 1b. Specifically, we utilize heterogeneous graph aggregation layers to disseminate and consolidate information throughout the AER Graph, resulting in the derivation of latent patient representations for ADR prediction. Simultaneously, we adopt HGT^[^ ^26^ ^]^ to construct default Heterogeneous Graph Convolution Layers. Additionally, we leverage contrastive learning techniques to enrich patient representations with an augmented view. The amalgamation of representations from the HGNNs and the augmentation view is subsequently employed by the Predictor to forecast potential ADRs.

PreciseADR excels in patient‐level ADR prediction as shown in Figure 1c,d. It consistently outperforms other baselines, underscoring its predictive capability in ADR detection and mitigation. Furthermore, PreciseADR surpasses baseline models in hit rate, as depicted in Figure 1d, establishing itself as a prominent advancement in ADR prediction. A higher hit rate indicates that PreciseADR more accurately identifies patient‐related ADRs, thus improving the quality of potential ADR predictions.

### 2.2 ADRs Related to Gender and Age

We identified ADRs related to gender and age using the PLEASE dataset comprising adverse event reports from the FAERS. It is widely acknowledged that ADRs are closely intertwined with the patient's individual health condition. Anderson et al.^[^ ^27^ ^]^ emphasize that ADRs observed in adults generally differ from those experienced by children. In addition, our research reveals ADRs that may exhibit underlying associations with patients' demographic characteristics, including age and gender. This groundbreaking discovery marks a significant paradigm shift in adverse event prediction, offering the potential to customize predictions for individual patients by leveraging their specific demographic information.

**Figure 2:**
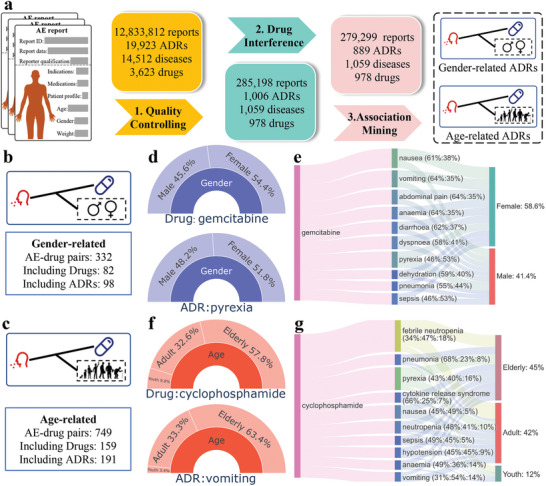
Gender‐related and age‐related Adverse Drug Reactions (ADRs) identified through our statistical methods. a) The pipeline of our approach uses adverse event reports to detect ADRs related to gender and age. Through this analysis, we successfully identified 98 significant ADRs related to gender and 191 related to age out of a total of 1266 ADRs. b,c, Statistics of detected ADRs related to gender (b) and age (c). d) Demographic information of “pyrexia” (a gender‐related ADR) and the drug “gemcitabine,” where the administration of ”gemcitabine” and the occurrence of “pyrexia” are more prevalent in females. e) Demographic information of “gemcitabine.” It is worth noting that males have a higher incidence of “pyrexia” when taking “gemcitabine.” f.) Demographic information of “vomiting” (an age‐related ADR) and the drug “cyclophosphamide,” where the consumption of “cyclophosphamide” or the occurrence of “vomiting” is more prevalent among elderly individuals than in adults. g) Demographic information of “cyclophosphamide.” It is noteworthy that among adults receiving “cyclophosphamide” and concurrently experiencing “vomiting,” their incidence surpasses that of elderly individuals.

The process of identifying ADRs associated with gender and age is illustrated in **Figure** **2**a, with detailed steps provided in Appendix A. To initiate this investigation, we assembled an extensive dataset comprising 12 million Adverse Event (AE) reports spanning the period from 2013 to 2022, sourced from FAERS. Subsequently, we subjected this dataset to rigorous curation through the “Quality Controlling” process, with a specific focus on incorporating high‐quality AE reports from reputable sources. Further refinement involved meticulous filtering to exclusively focus on AE reports directly linked to drug‐related events using the “Drug Interference” process. These carefully selected reports formed the basis for the subsequent “Association Mining” process. Through our systematic analysis, we identified a total of 98 gender‐related ADRs and 191 age‐related ADRs.

To provide further evidence of the association between ADRs and gender and age, Figure 2 details the demographic characteristics of patients reporting the gender‐related ADR “pyrexia” and the age‐related ADR “vomiting.” Specifically, the administration of “gemcitabine” (54.4% are females) and the occurrence of “pyrexia” (51.8% are females) are more prevalent in females, as illustrated in Figure 2d. It is noteworthy that among individuals receiving “gemcitabine” and concurrently experiencing “pyrexia,” 53% of them are males, exhibiting a higher incidence compared to their female counterparts, as shown in Figure 2e. Furthermore, although the consumption of “cyclophosphamide” (57.8% are elderly) or the occurrence of “vomiting” (63.4% are elderly) is more prevalent among elderly individuals compared to adults, as depicted in Figure 2f, it is noteworthy that 54% of those who are adults and receive “cyclophosphamide” while experiencing “vomiting” surpass the incidence rate of elderly individuals (14% of that population), as shown in Figure 2g.

### 2.3 Performance on the Patient‐Level ADR Prediction

PreciseADR demonstrates superior performance in patient‐level ADR prediction compared to baseline models. This innovative methodology leverages a wide array of patient‐specific data, including essential demographic attributes such as gender, age, and weight. Additionally, it delves into intricate medical histories and meticulously maintained medication records. This comprehensive approach ensures unparalleled accuracy in predictions, marking a significant stride toward patient‐centric healthcare. The emphasis on individualized risk assessments and preventative strategies reflects a holistic understanding of each patient's unique health profile, exemplifying the evolution of healthcare practices.

As outlined in **Table** **1**, the proposed PreciseADR framework stands out as the top performer among various baseline methods, including frequency‐based methods and deep learning methods. Note that frequency‐based methods predict ADRs based on the frequency of ADRs occurrence given indications (I) for diseases and given drugs (D). We specifically highlight a subset of deep learning methods referred to as “Given Drug” approaches, which primarily rely on drug‐related features for ADR prediction. These approaches offer insights into potential side effects at a broader drug level. The baselines with the “Given Drug” setting were constructed by exclusively utilizing drug features. Those baselines include MLP, Transformer,^[^ ^28^ ^]^ TabNet,^[^ ^29^ ^]^ AMFormer^[^ ^30^ ^]^ and GNN‐based methods. For GNN‐based methods, including GCN,^[^ ^31^ ^]^ GAT,^[^ ^32^ ^]^ GraphSAGE,^[^ ^33^ ^]^ GCNII,^[^ ^34^ ^]^ FANet,^[^ ^35^ ^]^ GATv2,^[^ ^36^ ^]^ and ARMA,^[^ ^37^ ^]^ a drug‐drug co‐occurrence graph^[^ ^38^ ^]^ was employed as the underlying graph structure to derive meaningful drug representations. The experimental results affirm that concentrating solely on drug side effects at this level already yields commendable outcomes. However, transitioning to ADR prediction at the individual patient level, incorporating patient‐specific features such as age, gender, medical history, and medication records, results in a notable 3.2% improvement w.r.t. AUC.

**Table 1:** Performances of PreciseADR and baselines on Patient‐level ADRs Prediction.

Type	Model	PLEASE		PLEASE‐Gender		PLEASE‐Age	
		AUC	Hit@10	AUC	Hit@10	AUC	Hit@10
Frequency	Random	0.5005	0.0317	0.5023	0.1957	0.5028	0.1085
	ADR‐Freq	0.5000	0.0825	0.5000	0.1836	0.5000	0.1289
	ADR‐Freq | I	0.5075	0.1956	0.5270	0.3216	0.5238	0.2727
	ADR‐Freq | D	0.5003	0.2084	0.5254	0.3354	0.5170	0.2835
	ADR‐Freq | I & D	0.5046	0.1748	0.5242	0.2905	0.5189	0.2431
Given Drug	MLP	0.7602	0.4115	0.7829	0.6548	0.7886	0.5535
	Transformer	0.7642	0.4691	0.7934	0.7093	0.7970	0.6287
	TabNet	0.5136	0.2753	0.5048	0.5156	0.5067	0.4368
	AMFormer	0.7748	0.4974	0.8060	0.7113	0.7999	0.6219
	GCN	0.7384	0.4599	0.7779	0.6718	0.7783	0.5906
	GAT	0.7544	0.4238	0.7832	0.6722	0.7849	0.5815
	GraphSAGE	0.7402	0.4535	0.7819	0.6791	0.7813	0.5757
	GCNII	0.7170	0.4706	0.7678	0.6949	0.7632	0.6123
	FANet	0.7148	0.4707	0.7685	0.7022	0.7593	0.6176
	GATv2	0.7614	0.4144	0.7816	0.6488	0.7879	0.5507
	ARMA	0.7197	0.4655	0.7613	0.6826	0.7312	0.6063
Patient‐level	PreciseADR	**0.8067**	**0.5431**	**0.8366**	**0.7541**	**0.8262**	**0.6776**

Another noteworthy observation within Table 1 pertains to the impact of incorporating information about drug‐drug co‐occurrence on predictive performance, particularly concerning Hit@10. In Table 1, all GNN‐based methods uniformly utilize MLP as the feature extractor and leverage a co‐occurrence graph of drugs to facilitate the learning of drug embeddings. This co‐occurrence graph is constructed by establishing edges between pairs of drugs whenever they jointly appear in an Adverse Event report. The inclusion of the drug‐drug co‐occurrence graph leads to a boost in predictive accuracy compared with MLP, particularly in terms of Hit@10, for all GNN models except GATv2. Another noteworthy observation within Table 1 pertains to the impact of incorporating information about drug‐drug co‐occurrence on predictive performance, particularly concerning Hit@10. In Table 1, all GNN‐based methods uniformly utilize MLP as the feature extractor and leverage a co‐occurrence graph of drugs to facilitate the learning of drug embeddings. This co‐occurrence graph is constructed by establishing edges between pairs of drugs whenever they jointly appear in an Adverse Event report. The inclusion of the drug‐drug co‐occurrence graph leads to a boost in predictive accuracy compared with MLP, particularly in terms of Hit@10, for all GNN models except GATv2. The reason why we use MLP to compare with GNN is that when GNN does not use graph structure, the effect of neural network forward propagation is equivalent to MLP. This phenomenon underscores the efficacy of incorporating additional information with graphs as a pivotal factor in enhancing the prediction capabilities of these GNN‐based models, consistently outperforming the traditional MLP and demonstrating competitive performance with Transformer. This also inspires us to incorporate the AER Graph into PreciseADR to enhance the model's performance.

**Figure 3:**
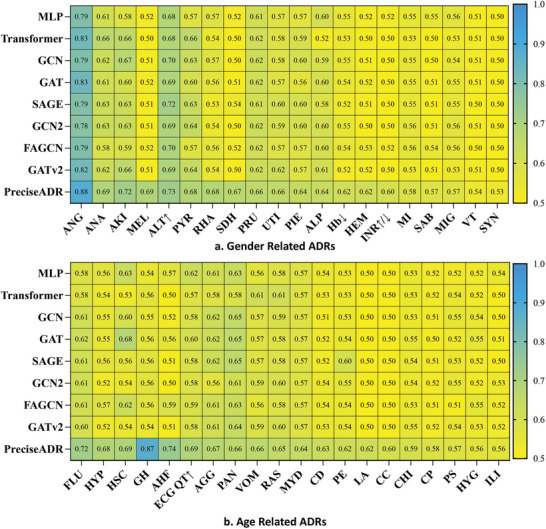
Performances of patient‐level ADR predictions on gender‐related ADRs (a) and age‐related ADRs (b). The proposed method PreciseADR also archives the best performances when predicting gender‐related and age‐related ADRs.

Furthermore, we conducted a thorough validation specifically targeting 20 Age‐related ADRs and 20 Gender‐related ADRs, as illustrated in **Figure** **3**. The outcomes of these experiments unequivocally underscore the robust performance of PreciseADR in both Age‐related ADRs and Gender‐related ADRs, with a majority of results surpassing those of the baseline methods.

### 2.4 Investigating Interpretability of PreciseADR

To further emphasize the significance of demographic information in patient‐level ADR prediction, we conducted an analysis to assess the impact of disturbing demographic‐related features on ADR predictions. We created three variants of the PreciseADR model, namely, “Disturb Gender,” which involved altering gender features for the testing data, “Disturb Age,” which perturbed age features, and “Disturb Gender & Age,” which perturbed both gender and age features.

**Figure 4:**
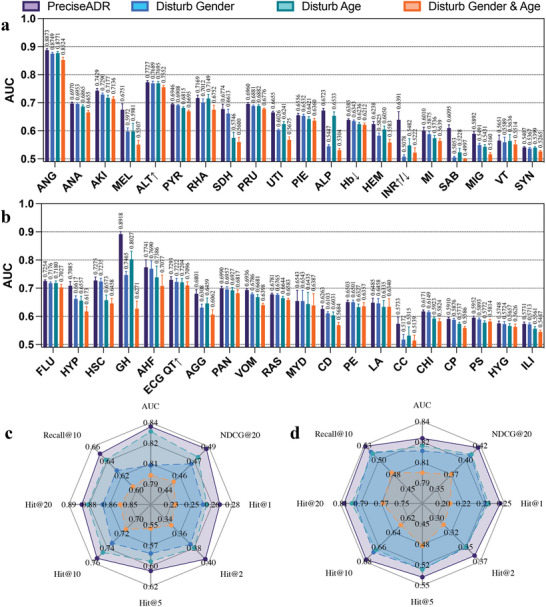
Performances of PreciseADR on gender‐related ADRs (a)(c) and age‐related ADRs (b)(d) with age and gender are disturbed. After perturbing the age and gender features, the performance of PreciseADR in predicting these ADRs experienced a decline. This suggests that both age and gender features have an impact on the accurate prediction of these ADRs.

The experimental results, as illustrated in **Figure** **4**, clearly demonstrate that introducing perturbations to both age and gender information results in a notable reduction in the model's prediction accuracy. It is worth highlighting that simultaneous perturbation of age and gender information has the most pronounced impact on the model's performance. This reaffirms the pivotal role of personal information in the ADR prediction process, underlining the necessity of considering such data for more accurate predictions. For example, when predicting the likelihood of a patient having “gastric haemorrhage” changing the patient's gender leads to a 14% decrease in the AUC, changing the patient's age results in a 9% decrease in the AUC, and simultaneously perturbing both gender and age causes a 26% reduction in AUC value.

### 2.5 Ablation Study

**Figure 5:**
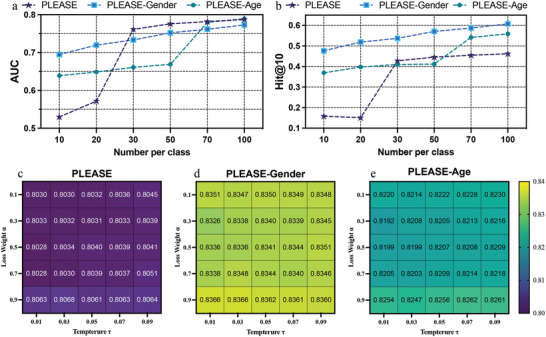
Ablation study on PreciseADR model w.r.t. training size (a and b), and contrastive learning module (c, d, and e). The increments of labeled data have the potential to significantly enhance the performance of the PreciseADR. And the introduction of contrastive learning has also improved the accuracy of the PreciseADR.

To further affirm the efficacy of our model design, we conducted additional ablation experiments, the results of which are presented in **Figure** **5**. The findings depicted in Figure 5a,b clearly illustrate that the performance of ADR prediction improves with an increase in the volume of labeled data. This underscores the importance of utilizing a larger quantity of labeled ADR records for model training to achieve superior results.

Furthermore, as shown in Figure 5c–e, the incorporation of a contrastive learning module significantly enhances the model's performance. Notably, it is observed that the optimal selection of hyperparameters for the model varies across different ADR prediction scenarios. This emphasizes the practical utility of employing automated machine learning techniques for optimal hyperparameter selection, thereby ensuring the model's effectiveness in diverse applications.

## 3 Discussion

This study commenced by identifying gender‐ and age‐related ADRs through statistical analyses, highlighting the significance of incorporating patient‐specific information for practical ADR prediction. In response to these findings, we introduced a patient‐level ADR prediction framework, PreciseADR, aimed at learning latent patient representations to enhance the precision of ADR predictions. PreciseADR achieves this by aggregating information from patients' physiological characteristics, medical histories, medication records, and ADRs. Our experimental results strongly support the effectiveness of our approach, demonstrating a significant performance improvement compared to conventional ADR prediction methods. Additionally, our experiments revealed that perturbing demographic features results in a reduction in PreciseADR's predictive performance, further emphasizing the importance of considering patient‐specific attributes in ADR prediction. This research carries substantial implications and holds a transformative impact on the precision medicine domain. It pioneers patient‐centric medicine, bolsters drug safety, and optimizes healthcare services. By focusing on Patient‐Level ADR prediction, PreciseADR enables tailored interventions and early ADR detection based on patient‐specific attributes and medical records.

While our approach effectively demonstrates its prowess in ADR prediction, it is crucial to acknowledge its inherent limitations. Primarily, our model predominantly relies on relatively broad patient attributes, encompassing parameters such as age, gender, medical history, and medication records. However, it refrains from incorporating finer‐grained omics data, such as scRNA‐seq data and scATAC‐seq data,^[^ ^39^, ^40^ ^]^ which has the potential to significantly enhance ADR prediction precision. The integration of genetic information, in conjunction with the established attributes, can provide a more comprehensive understanding of a patient's susceptibility to specific ADRs, ultimately refining predictive accuracy.

Another aspect warranting consideration pertains to the scalability of our approach. The inclusion of all ADR reports within the AER Graph may pose challenges as time advances, leading to the expansion of connections between Disease nodes, Drug nodes, and ADR nodes with the addition of more patient nodes. It is important to recognize that not all of these newly introduced patient nodes are indispensable and they might inadvertently introduce noise into the model. To address this issue, our future endeavors involve the utilization of explainable or causal inference methods.^[^ ^41^, ^42^ ^]^ Those approaches will serve the dual purpose of retaining critical patient data while concurrently identifying and isolating noisy patient data through anomaly detection. The ultimate objective is to enhance the robustness of ADR predictions and ensure the scalability and adaptability of the framework as it continues to evolve.

Furthermore, it is crucial to note that our method is primarily tailored for drug recommendation, focusing on predicting potential ADRs for drug combinations once the patient's disease is known. This approach inherently introduces a lag in predicting ADRs associated with new diseases and emerging drugs, as it relies on the availability of AE reports linked to specific drug‐disease combinations. To address this limitation, our future research endeavors are directed toward incorporating additional sources of information and pre‐trained models, such as molecular structures,^[^ ^43^, ^44^ ^]^ drug‐drug interactions.^[^ ^45^ ^]^ By expanding the scope of our approach in this manner, we aim to enhance our capacity for predicting ADRs related to new drugs and diseases, thus providing a more proactive and comprehensive prediction framework.

## 4 Related Works

Predicting ADRs associated with newly developed pharmaceuticals is pivotal for risk mitigation in novel drug trials. Various machine learning‐based methodologies for ADR predictions have been developed, including:

1) *Selected Feature‐based Approaches*: These methods predominantly utilize manually selected features (chemical attributes, textual descriptions, biomedical information) with machine learning or deep learning models for predicting adverse reactions.^[^ ^14^, ^15^, ^16^, ^17^, ^18^, ^19^, ^20^ ^]^ Additionally, the use of Simplified Molecular‐Input Line‐entry System (SMILES) for encoding chemical components is prevalent.^[^ ^23^, ^24^, ^25^ ^]^ For instance, SMILESConv^[^ ^23^ ^]^ employs a multi‐core convolutional network on SMILES sequences, while other studies^[^ ^24^ ^]^ transform SMILES tokens into a 2D molecular perspective with graph neural networks. These approaches heavily depend on chosen features, limiting adaptability to new settings.
2) *Drug Interaction‐based Approaches*: These methods primarily leverage drug interaction information to predict ADRs.^[^ ^46^, ^47^, ^48^ ^]^ For example, MARAS^[^ ^47^ ^]^ uses rule‐based data mining, and recent research^[^ ^48^ ^]^ leverages molecular‐level information and deep networks. These methods emphasize understanding drug interplay, not aligned with predicting ADRs in newly developed pharmaceuticals.
3) *Clinical Data‐based Approaches*: These methods utilize large‐scale clinical databases like Electronic Health Records (EHR) and healthcare claims data for ADR signal detection, relying on disproportionality analysis.^[^ ^49^, ^50^, ^51^ ^]^ Subsequent methodologies identify significantly correlated Drug‐ADR pairs and subsequently utilize machine learning techniques for predicting other drug‐related ADRs.^[^ ^5^, ^52^, ^53^, ^54^, ^55^ ^]^

The diverse methodologies employed address different aspects of ADR prediction, emphasizing the importance of considering various data sources, including patient‐specific information, for comprehensive and precise predictions. However, these methods do not take into account patient‐specific information and, therefore, fall short of achieving patient‐level ADR prediction. The proposed PreciseADR framework in this paper addresses this limitation by integrating patient information for more accurate ADR predictions.

## 5 Conclusion

In conclusion, PreciseADR introduces a pioneering framework for patient‐level Adverse Drug Reaction (ADR) prediction, contributing significantly to the progression of precision medicine. By unraveling the intricate network of associations among patients, drugs, diseases, and ADRs, PreciseADR surpasses conventional models, providing a holistic perspective in the healthcare domain. Its potential to enhance patient treatment outcomes and advance drug safety is both promising and expansive, positioning itself as a valuable tool for healthcare professionals in making informed, data‐driven decisions. As we gaze into the future, the integration of PreciseADR into clinical practices holds the potential to propel patient‐centered healthcare into its next evolutionary phase. It establishes the groundwork for safer and more personalized medical interventions, optimizing treatment outcomes while mitigating the risks associated with ADRs. Subsequent efforts for advancing PreciseADR involve the incorporation of additional expert knowledge, the application of interpretable machine learning approaches, and the integration of multimodal pre‐trained models into ADR prediction. This ongoing refinement aims to further enhance the precision, interpretability, and applicability of PreciseADR in diverse healthcare settings.

## 6 Experimental Section

### Dataset

A Patient‐LEvel Adverse drug reaction prediction dataset was curated, *PLEASE* for short, utilizing adverse event reports sourced from the FAERS.^[^ ^56^ ^]^ These FAERS reports contain crucial information, including demographic particulars (e.g., age and gender, devoid of personal identifiers), diseases, drug substances, and ADRs categorized as preferred terms in the MedDRA. Employing the data preprocessing procedures outlined in Appendix A, the PLEASE dataset was constructed, incorporating 279 299 adverse event reports, encompassing 889 unique ADRs, 1059 distinct diseases, and 978 diverse drugs. Furthermore, two specialized subsets were derived from the PLEASE dataset: PLEASE‐Gender and PLEASE‐Age, focusing on gender‐related and age‐related ADRs, respectively.

*Notation and representation of adverse event reports*. In this study, the PLEASE Dataset is represented as **X**_*P*_, where each element **x_i_** corresponds to a single patient safety report. The set of all diseases, medicines, and ADRs present in the dataset is denoted by D, M, and S, respectively. Each patient report is treated as a tuple that includes a set of diseases **d_i_**, a set of drugs **m_i_**, the patient's age *a*_*i*_, biological sex denoted by *g*_*i*_ (where one represents male and two represents female), weight *w*_*i*_, reporter's qualification *q*_*i*_, and reporting date *t*_*i*_. Thus, **x_i_** = (**d_i_**, **m_i_**, *a*_*i*_, *g*_*i*_, *w*_*i*_, *q*_*i*_, *t*_*i*_). The main objective was to predict the ADRs **s_i_** experienced by patient *i*, forming the label **y_i_** = **s_i_**. Given that a patient might take multiple medications simultaneously and experience several adverse drug events, each report contains a medicine set **m_i_** that is a subset of M, with each drug *m*_*j*_ ∈ **m_i_** represented by its DrugBank ID (string). Similarly, the disease set $d_{i}\subseteq D$ comprises one or more diseases, and the ADRs set $s_{i}\subseteq S$ consists of one or more drug side effects, with each *s*_*i*_ ∈ **s_i_** represented by its MedDRA ID. The patient's age, *a*_*i*_, is represented by an integer denoting the number of years, while the weight, *w*_*i*_, is represented by a real number in kilograms. The reporter's healthcare qualification, *q*_*i*_, falls into one of the five categories: physicians, pharmacists, other professionals, lawyers, and customers, denoted by integers 1–5, respectively.

### Problem Definition

The patient‐level ADR prediction problem could be formally defined as follows: Given a dataset of patient safety reports, represented as **X**_*P*_ = {**x_1_**, …, **x_P_**}, the objective is to construct a predictive model Predictor capable of accurately forecasting potential ADRs for individual patients **x_i_**, denoted as **y_i_** = Predictor(**x_i_**). Each patient **x_i_** ∈ **X**_*P*_ is characterized by their demographic and medical information, i.e., **x_i_** = (**d_i_**, **m_i_**, *a*_*i*_, *g*_*i*_, *w*_*i*_, *q*_*i*_, *t*_*i*_). The task entails predicting the ADRs **s_i_** experienced by each patient, thereby constituting the label **y_i_** = **s_i_**.

### Construction of Adverse Event Report Graph

An Adverse Event Report Graph (AER Graph) was introduced, denoted as G(N,E) to model and harness the interrelationships among patients, diseases, drugs, and ADRs, as depicted in Figure 1. The AER Graph served as a foundational component, facilitating precise prediction by capturing the intricate associations among patients $N_{P}$, diseases $N_{D}$, and medications $N_{M}$. The construction of the AER Graph represents a crucial preliminary step in comprehending and effectively dissecting ADRs. Within the structure of the AER Graph, individual nodes were employed to represent patients, diseases, and medications, while the edges denote connections between patients who have reported diseases and those who are prescribed specific drugs. More specifically, Bag‐of‐Words (BOW) features were employed to represent the node features of patients, denoted as **X**_*P*_. In contrast, the node features of diseases, **X**_*D*_, and medications, **X**_*M*_, were characterized using one‐hot features. It is important to note that the construction of the AER Graph exclusively relies on the training set of PLEASE.

### The neural architecture of PreciseADR

The PreciseADR framework, as illustrated in Figure 1, encompasses several key components, including Heterogeneous Graph Aggregation Layers, Patient Nodes Augmentation Layers, and the Predictor.

In the context of the AER Graph (G(N,E)), where node features belong to diverse types and domains, we initiate the process by projecting these features from their respective domains into a shared domain. The transformation is executed as follows:

(1) $$\begin{array}{ccc} H_{T}^{1} & = & \text{LayerNorm}\left(H_{T}^{0}W_{T}^{l}\right) \end{array}$$

where *T* ∈ {*P*, *D*, *M*} denotes the node type, $H_{T}^{0}$ represents the initial features, and *l* signifies the *l*‐th layer.

Subsequently, we employ Heterogeneous Graph Convolution Layers to facilitate the aggregation of messages within the AER Graph. The AER Graph is one of the heterogeneous graphs, consisting of diverse node types such as ADRs, Patients, and Drugs, along with a myriad of intricate relationships between them. The power of HGNNs was leveraged, which have demonstrated substantial capabilities in handling heterogeneous data structures. By modeling the diverse node types and their intricate relationships, HGNNs could effectively learn to aggregate messages along semantically meaningful meta‐paths, such as Patient→Drug→Patient and Patient→Disease→Patient. These paths capture important semantic associations, such as patients who have taken the same drugs or suffered from the same diseases, which can provide invaluable features for predicting patient‐level ADRs.

Here, HGT^[^ ^26^ ^]^ was used to build default Heterogeneous Graph Convolution Layers. The HGT architecture is designed to handle the inherent complexities of heterogeneous graphs, allowing for a more nuanced understanding of the relationships within the AER Graph. The procedure of each Layer of HGT can be broken down into the following core components:

(2) $$H^{l+1}\left[t\right]\leftarrow \underset{\forall s\in N(t),\forall e\in E(s,t)}{\text{Aggregate}}\left(\text{Attention}(s,e,t)\cdot \text{Message}(s,e,t)\right)$$

where *s* denotes the source node, *t* denotes the target node. There are three basic operators: Attention(*s*, *e*, *t*), which estimates the importance of each source node *s*; Message(·), which extracts the message by using the source node *s*; and Aggregate(·), which aggregates the neighborhood message for all source nodes *s* ∈ *N*(*t*) by the attention weight.

First, the attention was calculated, denoted by Attention(*s*, *e*, *t*), which signifies the importance of each source node *s* with respect to the target node *t*. This mechanism uses the Query–Value framework inspired by the Transformer architecture. The formula for attention calculation is:

(3) $$\begin{array}{ccc} \text{Attention}(s,e,t) & = & \underset{\forall s\in N(t)}{\text{Softmax}}\left(\underset{i\in [1,h]}{∥}ATT^{i}\left(s,e,t\right)\right) \end{array}$$

(4) $$\begin{array}{ccc} ATT^{i}\left(s,e,t\right) & = & \left(K^{i}\left(s\right)\;W_{\phi (e)}^{ATT}\;Q^{i}{\left(t\right)}^{T}\right)\cdot \frac{\mu_{\left\langle \tau (s),\phi (e),\tau (t)\right\rangle }}{\sqrt{d}} \end{array}$$

(5) $$\begin{array}{ccc} K^{i}\left(s\right) & = & {\text{K-Linear}}_{\tau (s)}^{i}\left(H^{\left(l\right)}\left[s\right]\right) \end{array}$$

(6) $$\begin{array}{ccc} Q^{i}\left(t\right) & = & {\text{Q-Linear}}_{\tau (t)}^{i}\left(H^{\left(l\right)}\left[t\right]\right) \end{array}$$

where τ(*t*) indicates the node type of node *t*, μ_〈τ(*s*), ϕ(*e*), τ(*t*)〉_ is a trainable vector that denotes the general significance of each meta‐relation triplet 〈τ(*s*), ϕ(*e*), τ(*t*)〉. Specifically, in the context of the *i*‐th attention head *ATT* ^*i*^(*s*, *e*, *t*), the transformation of the τ(*s*)‐type source node *s* into the *i*‐th *Key* vector *K* ^*i*^(*s*) is achieved through the application of a linear projection, denoted as ${\text{K-Linear}}_{\tau (s)}^{i}:R^{d}\to R^{\frac{d}{h}}$. Here, *h* represents the number of attention heads, and $\frac{d}{h}$ corresponds to the vector dimension per head. It is important to note that the indexing of ${\text{K-Linear}}_{\tau (s)}^{i}$ is based on the source node type τ(*s*). This implies that each type of node has a unique linear projection. This distinctive feature allows the model to adapt and tailor its projections according to the specific type of node, maximizing its ability to capture distribution differences within the data. This approach contributes to the model's capacity to effectively represent and understand heterogeneous information across different node types.

Once the attention was determined, the message was extracted, labeled as Message(*s*, *e*, *t*), based on the attention weight. The message is computed through a linear projection and includes source node *s* as well as edge dependency considerations. The target node *t* was projected as well with a linear projection ${\text{Q-Linear}}_{\tau (t)}^{i}$ into the *i* −th Query vector. The process is detailed as:

(7) $$\begin{array}{ccc} \text{Message}(s,e,t) & = & \underset{i\in [1,h]}{∥}MSG^{i}\left(s,e,t\right) \end{array}$$

(8) $$\begin{array}{ccc} MSG^{i}\left(s,e,t\right) & = & {\text{M-Linear}}_{\tau (s)}^{i}\left(H^{\left(l-1\right)}\left[s\right]\right)\;W_{\phi (e)}^{MSG} \end{array}$$

where ϕ(*e*) is the edge type of edge *e*. Specifically, to obtain the *i*‐th message head, *MSG* ^*i*^(*s*, *e*, *t*), we initiate the process by projecting the source node *s* of type τ(*s*) into the *i*‐th message vector. This projection was accomplished using a linear transformation, referred to as ${\text{M-Linear}}_{\tau (s)}^{i}:R^{d}\to R^{\frac{d}{h}}$. Here, *h* signifies the number of attention heads, and $\frac{d}{h}$ represents the vector dimension per head. Subsequently, a matrix denoted as $W_{\phi (e)}^{MSG}$ was introduced, with dimensions $R^{\frac{d}{h}\times \frac{d}{h}}$, to integrate edge dependency into the message representation.

The final step involves concatenating all *h* message heads to form the Message(*s*, *e*, *t*) for each node pair. Upon completing this process through a series of *L* Heterogeneous Graph Aggregation layers, the representation of patient nodes was obtained, denoted as $H_{P}^{L}$.

Subsequently, a fully connected layer was integrated, designated as the Patient Node Augmentation network, to layer $H_{P}^{S}$. This augmentation process introduced random noise to the original features during training, as defined by *Aug*(*X*) = *X* + (*E* − *Dropout*(*E*, ϵ)). However, during testing and prediction, dropout is not applied. Finally, a fully connected layer was used to predict the ADRs for each patient, expressed as $\tilde{Y}=\text{Predictor}\left(H_{P}^{L}+H_{P}^{S}\right)$. This comprehensive framework of PreciseADR enabled the effective aggregation of heterogeneous patient data, ultimately enhancing ADR predictions.

*Training objective*: The patient‐level ADR prediction task was framed as a multi‐class multi‐label classification problem, and Focal Loss^[^ ^57^ ^]^ was utilized as the training objective for dealing with imbalance ADRs, defined as:

(9) $$\begin{array}{c} L_{\text{focal}}=-{\left(1-Y\right)}^{\gamma }*log\left(\tilde{Y}\right). \end{array}$$

where *p* is the predicted probability of the correct class for a given sample. γ is the focusing parameter that controls the rate at which the loss decreases as the predicted probability *p* increases. γ can be adjusted to emphasize harder examples more (higher values) or to treat all examples equally (lower values).

Besides, the InfoNCE^[^ ^58^ ^]^ was applied to maximize the agreement between representations generated by the HGNN network and augmentation network and minimize the agreement between representations of unrelated data samples. The InfoNCE loss are as follows:

(10) $$\begin{array}{c} L_{\text{infonce}}=-\frac{1}{B}\sum_{i=1}^{B}\log\frac{\exp(\text{sim}\left(H_{P}^{L}\left[i\right],H_{P}^{S}\left[i\right]\right))}{\sum_{j=1}^{B}\exp\left(\text{sim}\left(H_{P}^{L}\left[i\right],H_{P}^{S}\left[j\right]\right)\right)} \end{array}$$

where *B* is the number of batch, *P* is the number of patient samples in the batch.

Therefore, the final training objective is as follows:

(11) $$\begin{array}{c} L=\alpha L_{\text{infonce}}+\left(1-\alpha \right)L_{\text{focal}} \end{array}$$

where α is a hyper‐parameter to adjust the weight of the InfoNCE loss.

### Data Availability

The data used in this paper, including the raw and processed adverse event report dataset, adverse event ontology, and drug ontology, are obtained from the research community via the project website at https://zitniklab.hms.harvard.edu/projects/patient‐safety. The raw adverse event reports are obtained from the FAERS. The raw adverse event ontology data from MedDRA are available at https://www.meddra.org/ , which requires subscriptions. The raw drug mapping data from DrugBank are available at https://go.drugbank.com/releases/latest.

## Conflict of Interest

The authors declare no conflict of interest.

## Author Contributions

Y.G. and X.Z. contributed equally to this work. H.W. conceived this study. H.W., X.Z., and Y.G. designed the method and drafted the manuscript. P.C. and X.Z. provided and preprocessed the data. X.Z. and Y.G. analyzed the experimental results and provided statistical analysis. Z.S. provided critical clinical insights. J.B. and X.Z. reviewed and revised the manuscript. H.W. coordinated and supervised the whole work.

## Data Availability

The data that support the findings of this study are available on request from the corresponding author.

## Appendix A The Construction of Please

In this study, we curated the Patient‐Level ADRs Prediction Dataset, known as PLEASE. To ensure the inclusion of high‐quality adverse event (AE) reports, we established a rigorous three‐stage data processing pipeline, comprising data acquisition, quality control, and consideration of drug interference.

**Data Acquisition**: Our journey commenced with the acquisition of raw data from publicly accessible sources. The primary source of our adverse event reports was the FAERS. FAERS stands as a pivotal platform for post‐marketing pharmacovigilance. The reports contained vital demographic information, encompassing details like age and gender (without any personal identifiers), records of drug substances, and descriptions of ADRs, categorized as preferred terms following the Medical Dictionary for Regulatory Activities (MedDRA). In total, our investigation encompassed a vast dataset of 10 443 476 reports. These reports documented 19 193 distinct ADRs and were associated with 3624 unique drugs, spanning the period from January 2013 to September 2022. To enhance the utility of this data, we meticulously linked adverse event descriptors to their respective MedDRA ID preferred terms and further mapped them to human organ systems utilizing the MedDRA ontology. Similarly, drug descriptors were mapped to DrugBank IDs and categorized based on the Anatomical Therapeutic Chemical (ATC) classification system.

**Quality Control**: To uphold the integrity and reliability of our training data, we executed a series of quality control measures:

1) In instances where multiple reports shared the same case number, we retained only the most recent report, ensuring the latest and most relevant information.
2) Our analysis was confined to ADRs occurring within the United States. This approach aimed to circumvent biases arising from different national surveillance systems and country‐specific variations.
3) We concentrated on reports submitted by healthcare professionals, as they are more likely to possess the necessary domain expertise to accurately distinguish true adverse drug reactions from indications or unrelated symptoms.
4) To ensure ample historical data for robust model training and prediction, we retained diseases, medications, and adverse event types that appeared in the dataset more than 100 times.
5) We retained reports that contained information on diseases, medications, and ADRs, with none of these fields being empty. This step aimed to enhance the completeness and reliability of our dataset for subsequent analysis.

**Drug Interference**: ADRs can encompass various issues such as adverse reactions, side effects, medication errors, infections, surgical complications, device malfunctions, and more, all of which can have negative impacts on a patient's health. In this work, we only concentrate on ADRs related to drugs, including adverse reactions and side effects. Therefore, we apply drug interference after quality control, to find ADRs related to drugs.

After drug interference, all the filtered AE reports are used to build PLEASE. Furthermore, we applied the “association mining” stage to identify Gender‐related and Age‐related ADRs with PLEASE.

**Association Mining** Intuitively, a patient's gender, age, and other personal characteristics can influence the prognosis and the likelihood of experiencing certain ADRs. In this study, we discovered these facts through statistical methods and identified ADRs that are correlated with gender and age. Specifically, we assessed the significance of the gender/age—ADR associations by one‐sided Fisher test, and his option returns a significant p–value only in the event of a positive association.

We use gender‐related ADRs and age‐related ADRs to build PLEASE‐Gender and PLEASE‐Age. The statistical information of the PLEASE dataset and its variants are as shown in **Table** **A1**.

**Table A1:** Statistics of the PLEASE dataset and its variants.

Datasets	# Patients	# ADRs	# Drugs	# Train data	# Val data	# Test data
PLEASE	279 299	889	978	209 475	34 912	34 912
PLEASE‐Gender	165 734	83	978	124 300	20 717	20 717
PLEASE‐Age	199 413	184	978	149 559	24 927	24 927

## Appendix B Variants of PreciseADR

This study introduces a patient‐level ADR prediction framework PreciseADR, with HGT (Heterogeneous Graph Transformer) being the default backbone. In this section, variants models of PreciseADR were employed for patient‐level ADR prediction, broadening the scope of the investigation and enhancing the comparative analysis of predictive methodologies at the patient level. Those variants including using other models as backbone, including MLP, Transformer, GCN,^[^ ^31^ ^]^ GAT,^[^ ^32^ ^]^ GraphSAGE,^[^ ^33^ ^]^ GCNII,^[^ ^34^ ^]^ FANet,^[^ ^35^ ^]^ GATv2,^[^ ^36^ ^]^ RGCN.^[^ ^59^ ^]^ The performances of PreciseADR variants are shown in **Table** **A2**. In the comparative analysis of GNN‐based methods, the integration of the AER Graph proves notably effective in capturing the intricate relationships between patients and Adverse Drug Reactions (ADRs), leading to enhanced precision in ADR prediction. Furthermore, the assessment of Heterogeneous Graph Neural Network (HGNN)‐based versus traditional GNN‐based methods highlights the superior performance of models adept at handling heterogeneous information. This underscores the importance of introducing the AER Graph and utilizing HGNN as the main component of the PreciseADR framework. The comprehensive analysis conducted herein illuminates that the incorporation of traditional models into the PreciseADR framework leads to substantial performance improvements. The synergistic integration of established methodologies with the precision‐driven ethos of PreciseADR is vividly depicted in the results, emphasizing the intrinsic value of this fusion. These findings mark a significant advancement in the landscape of ADR prediction, showcasing the instrumental role of traditional models in augmenting the predictive capabilities of the PreciseADR paradigm.

**Table A2:** Results of PreciseADR variants with other models replace Heterogeneous Graph Aggregation Layers.

Variants	PLEASE		PLEASE‐Gender		PLEASE‐Age	
	AUC	Hit@10	AUC	Hit@10	AUC	Hit@10
PreciseADR‐MLP	0.7787	0.4971	0.8030	0.7239	0.8031	0.6436
PreciseADR‐Transformer	0.7880	0.5223	0.8143	0.7428	0.8171	0.6648
PreciseADR‐GCN	0.7758	0.4985	0.7978	0.7292	0.8003	0.6488
PreciseADR‐GAT	0.7952	0.5289	0.8171	0.7493	0.8184	0.6708
PreciseADR‐GraphSAGE	0.7990	0.5318	0.8215	0.7495	0.8202	0.6718
PreciseADR‐GCNII	0.8008	0.5362	0.8251	0.7541	0.8248	0.6773
PreciseADR‐GATv2	0.7947	0.5317	0.8192	0.7486	0.8223	0.6724
PreciseADR‐FANet	0.7878	0.5141	0.8093	0.7405	0.8116	0.6617
PreciseADR‐RGCN	0.8038	0.5345	0.8277	0.7572	0.8264	0.6776
PreciseADR‐HGT	0.8067	0.5431	0.8366	0.7541	0.8262	0.6776

## Appendix C ADR Abbreviation

In this section, we present **Table** **A3**, which delineates the abbreviations for ADRs used in Figures 3 and 4, mapped to their corresponding ADRs. The table is meticulously structured to enhance readability and facilitate quick reference. To optimize its utility, the table is divided into two distinct sections: the left two columns display gender‐related ADR abbreviations, while the right two columns are dedicated to age‐related ADR abbreviations. For instance, gender‐related ADRs such as “Ventricular Tachycardia (VT)” and “Migraine (MIG)” are grouped separately from age‐related ADRs like “Acute Kidney Injury (AKI)” and “Gastric Hemorrhage (GH).”

**Table A3:** The ADR Abbreviations used in Figure 4.

Abbr.	ADR	Abbr.	ADR
ANG	Angioedema	FLU	Flushing
ANA	Anaemia	HYP	Hyperkalemia
AKI	Acute Kidney Injury	HSC	Hospice Care
MEL	Melaena	GH	Gastric Haemorrhage
ALT↑	Alanine Aminotransferase Increased	AHF	Acute Hepatic Failure
PYR	Pyrexia	ECG QT↑	Electrocardiogram QT Prolonged
RHA	Rhabdomyolysis	AGG	Aggression
SDH	Subdural Haematoma	PAN	Pancreatitis
PRU	Pruritus	VOM	Vomiting
UTI	Urinary Tract Infection	RAS	Rash
PIE	Pain in Extremity	MYD	Mydriasis
ALP	Alopecia	CD	Chest Discomfort
Hb↓	Hemoglobin Decreased	PE	Pulmonary Embolism
HEM	Haematuria	LA	Lactic Acidosis
INR↑/↓	International Normalized Ratio Abnormal	CC	Circulatory Collapse
MI	Myocardial Infarction	CHI	Chills
SAB	Spontaneous Abortion	CP	Chest Pain
MIG	Migraine	PS	Peripheral Swelling
VT	Ventricular Tachycardia	HYG	Hyperglycemia
SYN	Syncope	ILI	Influenza‐like Illness
